# Loss of the oncogenic phosphatase PRL-3 promotes a TNF-R1 feedback loop that mediates triple-negative breast cancer growth

**DOI:** 10.1038/oncsis.2016.50

**Published:** 2016-08-15

**Authors:** H H Gari, G D DeGala, M S Lucia, J R Lambert

**Affiliations:** 1 Department of Pathology, University of Colorado Anschutz Medical Campus, Aurora, CO, USA

## Abstract

Stimulating tumor cell senescence and apoptosis are proven methods for therapeutically combating cancer. However, senescence and apoptosis are conventionally viewed as parallel, not sequential, processes. We have discovered that the metastasis-promoting phosphatase, PRL-3, is transcriptionally regulated by the NF-ĸB pathway in triple-negative breast cancer (TNBC) cells, and that PRL-3 knockdown elicits an autocrine tumor necrosis factor receptor 1 (TNF-R1) feedback loop that results in TNBC cell senescence followed by apoptosis. Knockdown of PRL-3 leads to rapid G1 cell cycle arrest and induction of a strong TNFα cytokine response that promotes a period of cellular senescence through TNF-R1-mediated activation of NF-ĸB. Senescent PRL-3 knockdown cells subsequently underwent apoptosis as a result of increased TNF-R1 signaling through the TNFα-associated extrinsic death pathway, shunting signaling away from the NF-ĸB cascade. These data suggest that TNF-R1 signaling dynamically re-programs after PRL-3 knockdown, from sustaining cell senescence through NF-ĸB to promoting apoptosis through TNF-R1 internalization and caspase-8 activation. The molecular mechanisms that determine the survival–death balance of TNF-R1 signaling are poorly understood, despite the fact that TNF-R1 has been extensively studied. Our results describe PRL-3 knockdown as a novel survival–death balance modifier of the TNF-R1 pathway, and show that senescent TNBC tumor cells can be sensitized to undergo apoptosis in a sequential manner.

## Introduction

Breast cancer is the most commonly diagnosed cancer and principal cause of cancer-related mortality in women worldwide.^1^ Owing to advancements in high-throughput gene expression profiling, breast cancer has been clustered into five major subtypes based on estrogen receptor (ER) expression, progesterone receptor expression and human epidermal growth factor receptor 2 (HER2) amplification.^2^ Several anti-hormonal therapies are FDA-approved for breast cancer patients with tumors expressing ER or progesterone receptor, while targeted therapy with the monoclonal antibodies trastuzumab and pertuzumab are indicated for patients with tumors exhibiting HER2 amplification. This categorization system, based on hormone receptor and HER2 status and the subsequent coupling of anti-hormonal and HER2 targeted therapy, is one of the first examples in modern oncology for molecular subtyping and personalized treatment that has resulted in significant decreases in disease burden and overall mortality.

Triple-negative breast cancers (TNBCs), which comprise 15–20% of all newly diagnosed cases of breast cancer, lack expression of ER, progesterone receptor and amplification of HER2 and are rapidly progressive; typically, they are diagnosed as high grade tumors that are invasive by the time of diagnosis.^3^ Because TNBCs lack expression of ER, progesterone receptor and HER2 amplification, cytotoxic chemotherapies are most frequently utilized.^4, 5^ However, these treatments are limited, particularly in the unselected metastatic population, by poor long-term therapeutic response, non-selective toxicities and clonal progression of disease with the development of resistance. Thus, there is a vital unmet need to understand molecular processes that promote the aggressive nature of TNBC, and a need to identify novel mechanisms for enhancing cancer cell death so that new therapeutic strategies may be explored.

We previously reported on a genome-wide functional genetic shRNA screen conducted in our laboratory to identify genes that, when silenced, conferred resistance to the anticancer agent, AMPI-109.^6^ The highest ranking hit from our screen was the metastasis-promoting phosphatase, phosphatase of regenerating liver (PRL-3). We identified PRL-3 as a protein tyrosine phosphatase amplified or upregulated in approximately 19–31% of invasive basal breast cancers.^6^ Though TNBC and basal breast cancers are not equivalent, there is considerable overlap. Up to 55% of basal-like breast cancers are triple-negative, and up to 65% of TNBCs are basal-like.^7^ In our validation experiments, we demonstrated that PRL-3 knockdown resulted in substantial growth inhibition and significantly impaired the migratory and invasive ability of TNBC cells.^6^ These studies, which have been independently verified,^8^ establish a strong case for the investigation of PRL-3 as an oncogene in TNBC. However, elucidation of the exact mechanisms by which loss of PRL-3 expression impairs TNBC growth remains poorly understood.

Tumor necrosis factor alpha (TNFα) is a pleiotropic cytokine that binds tumor necrosis factor receptor 1 (TNF-R1) and elicits diverse responses ranging from maintaining cell viability and proliferation to activation of apoptosis.^9, 10^ Upon TNFα binding, TNF-R1 recruits the adaptor, TRADD, to its cytoplasmic death domain.^11, 12^ TRADD acts as a scaffolding platform to recruit both RIP-1 and TRAF-2 to activate either the nuclear factor-kappa B (NF-ĸB) or activator protein 1 (AP-1) pathway,^11^ or recruits FADD and pro-caspase-8 to initiate apoptosis.^13, 14^ Little is known about what regulates this survival–death balance of TNF-R1 signaling, despite the fact that it has been intensely studied since the 1980s.

In this report, we demonstrate that PRL-3 is transcriptionally regulated by the pro-inflammatory NF-ĸB pathway in TNBC cells, and that PRL-3 knockdown elicits an autocrine TNF-R1 feedback loop that results in cell cycle arrest and senescence as a pre-determinant to engaging apoptosis of TNBC cells. These studies reveal a previously undescribed mechanism for how PRL-3 influences TNBC cell growth and further increase our understanding of the role of TNF signaling in the disease.

## Results

### Knockdown of PRL-3 induces senescence in TNBC cell lines

**Figure 1:**
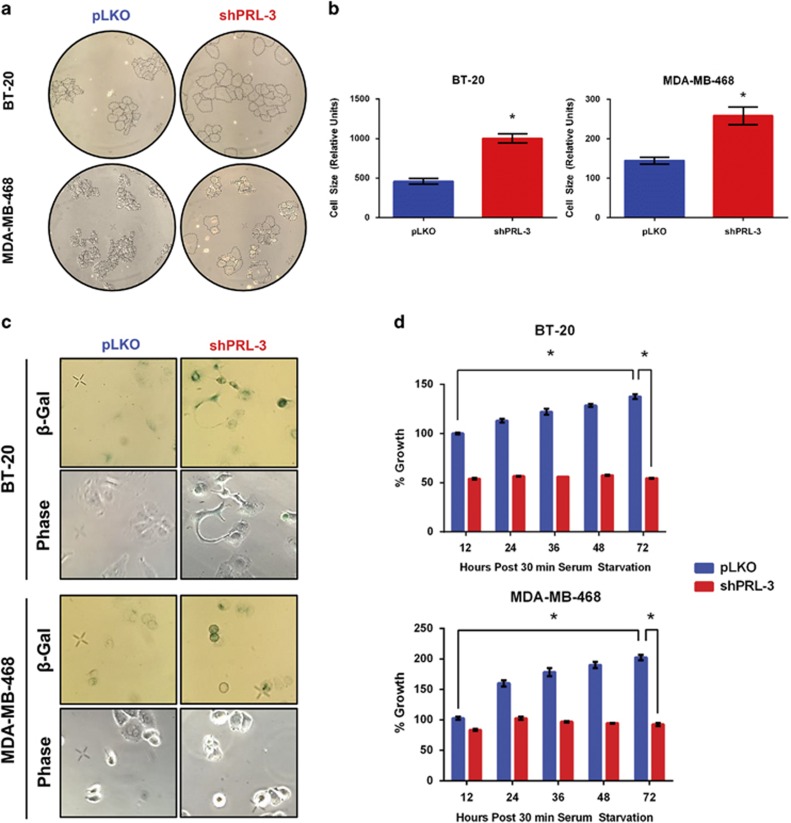
Knockdown of PRL-3 induces senescence in TNBC cell lines. (**a**) Phase contrast images of BT-20 and MDA-MB-468 cells transduced with lentivirus to knockdown PRL-3 (shPRL-3 #1) or non-silencing control (pLKO). (**b**) Quantification of (**a**) as determined by ImageJ analysis. (**c**) Phase contrast images depicting SA-β-Gal-positive cells after PRL-3 knockdown in BT-20 and MDA-MB-468 TNBC cells. (**d**) Each bar depicts the effect of increased serum concentration (20% fetal bovine serum (FBS) vs 10% FBS) on cellular growth as assessed by MTS assay on PRL-3 knockdown, SA-β-Gal-positive clones (red bars). Control (pLKO) cell growth in depicted by blue bars. Images captured at × 10 magnification. Data represented are the mean±s.d. of three independent experiments. *P<0.05 as determined by Student *t*-test on final data point.

Previously, we reported on the role of PRL-3 on impairing the growth of TNBC cells.^6^ Here, we analyzed changes in cellular morphology as a visual indicator of cell viability following stable lentiviral knockdown of PRL-3 in two TNBC cell lines, BT-20 and MDA-MB-468, in an effort to identify molecular mechanisms of PRL-3-mediated growth inhibition. Knockdown of PRL-3 was associated with substantial increases in cellular size, accompanied by a spreading morphology in both TNBC cell lines (Figures 1a and b).

Enlargement of cell size is considered to be an indicator that cells may be engaging a senescence program.^15^ To further test the hypothesis that cells with PRL-3 knocked down enter senescence, we examined PRL-3 knockdown cells for the expression of senescence-associated-β-galactosidase (SA-β-Gal) at pH 6.0, a hallmark of senescent cells. Knockdown of PRL-3 was associated with increased cytoplasmic SA-β-Gal staining in both TNBC cell lines (Figure 1c), with minimal SA-β-Gal staining observed in control cells. We also examined the ability of mitogenic stimulation to restore cellular growth in SA-β-Gal-positive clones, as senescent cells should not respond to growth factor stimulation. PRL-3 knockdown SA-β-Gal-positive cells did not respond to increased mitogenic stimulation (20% fetal bovine serum) relative to control cells over 72 h (Figure 1d). Taken together, these data indicate that knockdown of PRL-3 in TNBC cells leads to initiation of an irreversible cell senescence program.

### Senescent PRL-3 knockdown TNBC cells arrest in G1 despite differences in checkpoint aberrations

**Figure 2:**
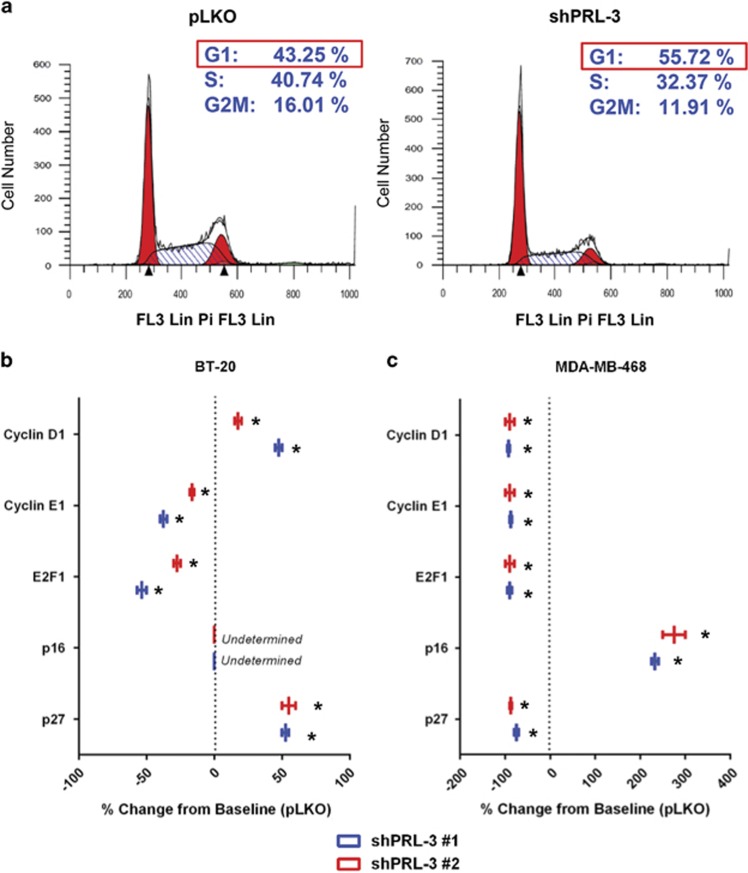
Senescent BT-20 cells with PRL-3 knockdown arrest in G1 despite unique cell cycle checkpoint deletions. (**a**) Flow cytometry assessment of DNA quantitation using propidium iodide incorporation in BT-20 control (pLKO) and PRL-3 knockdown (shPRL-3 #1) cells. (**b**, **c**) qRT–PCR results depicting percent RNA change of cell cycle checkpoint proteins in BT-20 and MDA-MB-468 cells, respectively, transduced with two shRNA clones to PRL-3 (shRNA #1 (blue) and shRNA #2 (red)). Data are represented as percent change from control (pLKO) expression. Data represented are the mean±s.d. of three independent experiments. *P<0.05 as determined by Student *t*-test.

Basak and colleagues^16^ previously reported that PRL-3 enforces G1 cell cycle arrest in triple knockout mouse embryonic fibroblasts deficient for all three retinoblastoma protein (Rb) family members (RB1, p107 and p130). To determine the phase of the cell cycle in which PRL-3 knockdown induces arrest in TNBC cells, we assessed DNA content using propidium iodide and flow cytometry. An increase in the G1 population of approximately 12.5% was observed following PRL-3 knockdown in BT-20 cells (Figure 2a). These data augment a previously reported finding that knockdown of PRL-3 in TNBC cells can lead to a G1 block.^8^

To identify whether PRL-3 knockdown results in G1 arrest pre- or post-restriction point (R_pt_), however, we carried out mRNA molecular marker analyses for several cyclin-dependent kinase inhibitors, including p16^INK4A^ (pre-R_pt_) and p27 (post-R_pt_), and cyclins D1 (pre-R_pt_) and E1 (post-R_pt_).

Following PRL-3 knockdown in BT-20 cells, which possess a homozygous deletion for p16^INK4A^, we observed upregulation of p27 at the RNA level (Figure 2b). We also observed an increase in cyclin D1 levels, indicating BT-20 cells are capable of progressing through the R_pt_ as a consequence of the p16^INK4A^ deletion, which relieves checkpoint repression.

In contrast, MDA-MB-468 cells, which contain a partial homozygous deletion for RB1, upregulate p16^INK4A^ to induce G1 arrest after PRL-3 knockdown (Figure 2c). These data suggest that in contrast to BT-20 cells, arrest in MDA-MB-468 cells occurs pre-R_pt_, and may prevent the cell from benefiting from relieved repression of E2F1 as a result of the RB1 deletion. These data reveal that PRL-3 knockdown induces G1 arrest in TNBC cells irrespective of cyclin-dependent kinase inhibitor aberrations that normally create a permissive environment for G1/S transition.

### NF-ĸB binds the PRL-3 promoter and regulates PRL-3 expression

We reasoned that if PRL-3 knockdown associates with cell cycle arrest and senescence in TNBC cells, then understanding regulators of PRL-3 could provide clues toward PRL-3 function or pathway association. To this end, we carried out microarray co-expression analyses of genes that clustered with PRL-3 expression across multiple TNBC cell lines using the GENE-E algorithm (www.broadinstitute.org). Through this analysis, we identified an upstream effector member of the NF-ĸB pathway, TRAPPC9, as a gene with expression levels that track positively with PRL-3 expression (r=0.692, data not shown).

NF-ĸB is a widely expressed transcription factor that regulates the expression of numerous genes involved in inflammatory responses, cell growth, differentiation and apoptosis.^17, 18^ NF-ĸB is a dimer of members of the Rel family of proteins each containing a highly conserved amino-terminal DNA-binding/dimerization domain called the Rel homology domain. This region also contains a nuclear localization signal critical for NF-ĸB activity. The NF-ĸB/Rel family includes NF-ĸB1 (p50/p105), NF-ĸB2 (p52/p100), RelA (p65), RelB and cRel.^19^ Owing to the presence of a strong transcriptional activation domain, p65 is responsible for most of NF-ĸB transcriptional activity with the most prevalent activated form of NF-ĸB being a heterodimer consisting of p50 and p65.^20^

**Figure 3:**
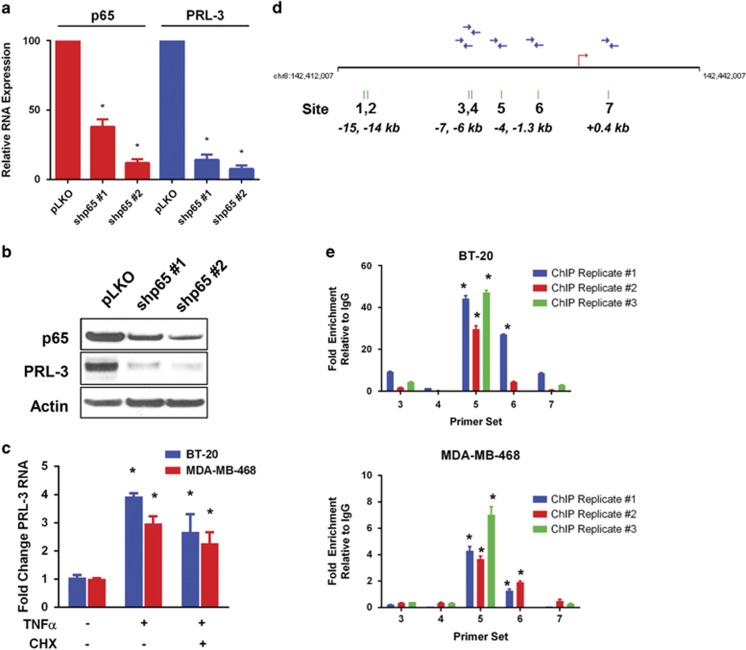
The NF-ĸB transcription factor subunit, p65, binds the PRL-3 promoter and regulates PRL-3 expression. (**a**) BT-20 RNA levels as assessed by qRT–PCR for p65 (red) and PRL-3 (blue) following p65 knockdown using two different shRNA clones (shp65 #1 and shp65 #2). (**b**) Immunoblot analysis of p65 and PRL-3 protein levels following p65 knockdown in BT-20 cells. (**c**) Fold change in PRL-3 RNA in BT-20 (blue) and MDA-MB-468 (red) cells as determined by qRT–PCR after 6 h treatment with TNFα (20 ng/ml) or TNFα (20 ng/ml) and cycloheximide (CHX, 100 nM). (**d**) Schematic of the human PRL-3 promoter locus. Seven potential NF-ĸB-binding sites indicated with green hash marks and numbered, with the position of each site with respect to the transcription start site (red arrow) indicated. Blue arrows represent qRT–PCR oligonucleotide primers designed to recognize the respective binding sites. (**e**) Results of three independent replicate chromatin immunoprecipitation experiments performed in BT-20 and MDA-MB-468 cells following 30 min treatment with 20 ng/ml TNFα. Data are represented as fold enrichment relative to IgG control. Data represented are the mean±s.d. of three independent experiments. *P<0.05 as determined by Student *t*-test.

Because TRAPPC9 is transcriptionally regulated by p65, we presumed that PRL-3, may also be regulated by p65 owing to similarities in expression patterns. We examined this by knocking down p65 using two shRNA clones and observed decreased PRL-3 RNA (Figure 3a) and protein (Figure 3b), suggesting p65 may also be involved in regulating PRL-3 expression.

To further test this hypothesis, we examined PRL-3 RNA levels following treatment with recombinant human TNFα to activate the NF-ĸB pathway, and observed a threefold to fourfold increase in PRL-3 RNA in two TNBC cell lines (Figure 3c). To determine whether PRL-3 is a direct transcriptional target for p65, we co-treated cells with TNFα and cycloheximide to block *de novo* protein synthesis and observed a 2.5–3-fold increase in PRL-3 RNA demonstrating that transcriptional activation of PRL-3 by p65 is direct (Figure 3c).

To determine whether p65 can bind the PRL-3 promoter, we first analyzed a 20 kb genomic region spanning the PRL-3 transcriptional start site using the University of California Santa Cruz genome browser database (http://genome.ucsc.edu) and identified seven potential NF-ĸB-binding elements (Figure 3d). We examined p65 binding *in vivo* by performing chromatin immunoprecipitation followed by qRT–PCR using oligo primers designed to recognize each of the five consensus sites closest to the transcriptional start site (Figure 3d, sites 3–7; blue arrows). In BT-20 cells, we observed a 30–40-fold enrichment of p65 binding following TNFα treatment at consensus site 5, with minimal loading at sites 3, 6 and 7 in BT-20 cells (Figure 3e). Similarly, we observed a fourfold to sevenfold enrichment of p65 binding in MDA-MB-468 cells at site 5, and minimal loading at site 6 (Figure 3e). These data suggest that in response to TNFα, p65 largely, and directly, regulates PRL-3 RNA production in TNBC cells.

### TNBC cells with PRL-3 knocked down upregulate TNFα leading to activation of NF-ĸB during the senescence period

In most cells, NF-ĸB exists in the cytoplasm in an inactive form associated with regulatory proteins termed inhibitors of ĸB (IĸB). In the absence of stimulation, the interaction between NF-ĸB and IĸBs prevents the nuclear localization signal in NF-ĸB from being recognized, preventing its nuclear localization. The majority of signals that lead to the activation of NF-ĸB result in activation of a high molecular weight complex containing a serine-specific IĸB kinase (IKK). Phosphorylation of IĸB by IKK targets IĸBs for ubiquitin-dependent degradation by the proteasome.^21^ Degradation of IĸBs results in unmasking of the nuclear localization signal in NF-ĸB, translocation of NF-ĸB to the nucleus and activation of NF-ĸB target genes.

**Figure 4:**
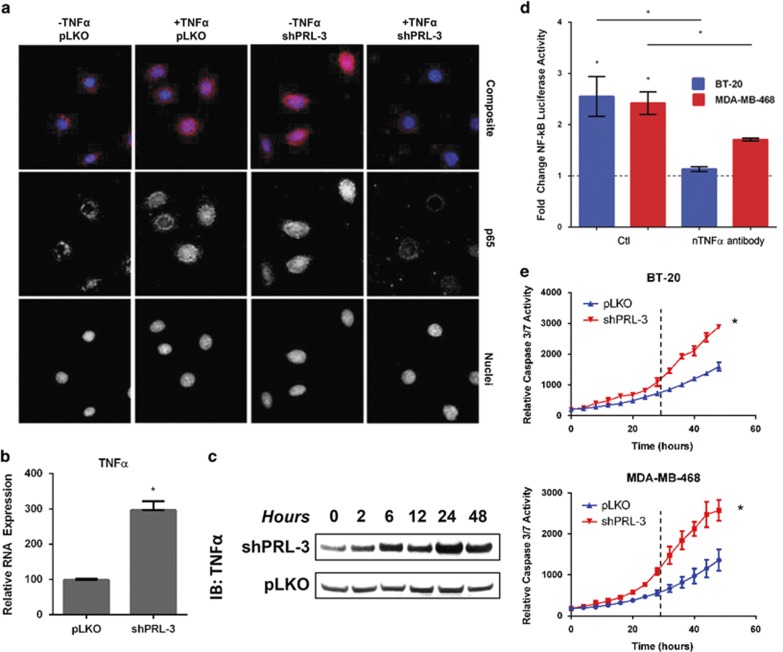
Continued suppression of PRL-3 expression results in a TNFα-associated apoptosis feedback loop in TNBC cell lines. (**a**) Composite immunofluorescence images (top row) of p65 (middle row) and DAPI-stained nuclei (bottom row) following PRL-3 knockdown and treatment with 20 ng/ml TNFα in BT-20 cells. Images were captured at × 100 magnification. (**b**) Expression level of TNFα RNA at 24 h following PRL-3 knockdown in BT-20 cells as assessed by qRT–PCR. (**c**) Immunoblot analysis of secreted TNFα protein over the course of 48 h following PRL-3 knockdown in BT-20 cells. (**d**) Fold change of NF-ĸB activity in BT-20 (blue) and MDA-MB-468 (red) cells exhibiting PRL-3 knockdown, as assessed by transfection with an NF-ĸB-luciferase reporter plasmid. Cells were treated with a control IgG antibody (Ctl) or a neutralizing TNFα antibody (nTNFα antibody). Values were normalized to those from pLKO control cells. (**e**) Real-time kinetic monitoring of caspase-3/7 activity in control (blue) and PRL-3 knockdown (red) BT-20 and MDA-MB-468 cells as quantified by the IncuCyte Zoom Live Cell Imaging System. Data represented are the mean±s.d. of three independent experiments. *P<0.05 as determined by Student *t*-test. IB, immunoblot.

p65 target genes, including IĸBs, are known to exert reciprocal regulation on the NF-ĸB pathway.^21^ To assess whether PRL-3 similarly functions as part of a feedback loop, we determined the effect of PRL-3 knockdown on the ability of p65 to translocate to the nucleus. We hypothesized that if PRL-3 knockdown impaired the growth of TNBC cells, p65 may be restricted from nuclear translocation and driving expression of pro-survival genes. Following PRL-3 knockdown in BT-20 cells, we made the surprising observation that p65 localized to the nucleus in the absence of TNFα treatment (Figure 4a), suggesting activation of NF-ĸB. Furthermore, knockdown of PRL-3 and treatment with exogenous TNFα was associated with diminished p65 staining, suggesting possible downregulation of the pathway.

To determine whether nuclear p65 following PRL-3 knockdown was attributed to an increase in TNFα, we examined TNFα mRNA and protein in BT-20 cells with PRL-3 knockdown. We observed a significant increase in TNFα RNA (Figure 4b) and protein (Figure 4c). Moreover, this increase in TNFα was capable of activating the NF-ĸB pathway in an autocrine manner, as determined by transiently transfecting PRL-3 knockdown cells with an NF-ĸB luciferase reporter. We observed a ~2.5-fold induction of luciferase activity (Figure 4d), which was largely reversible using a TNFα-neutralizing antibody in the culture media.

### Continued suppression of PRL-3 results in a dynamic TNFα-associated apoptosis feedback loop in senescent TNBC cell lines

As upregulation of TNFα and activation of NF-ĸB is associated with the senescence period in PRL-3 knockdown cells, we hypothesized that eventually, PRL-3 knockdown cells may become sensitized to increased levels of TNFα, leading to apoptosis. We examined apoptosis by kinetic monitoring of caspase-3/7 activity and observed that PRL-3 knockdown cells underwent a higher rate of apoptosis relative to control cells 24 h after transduction (Figure 4e). The timing of this higher apoptosis rate coincides with the approximate time TNFα levels are highest following PRL-3 knockdown (Figure 4c; 24 h). Prior to 24 h, the rate of apoptosis is equivalent to control cells, suggesting PRL-3 knockdown cells are senescent and remain viable.

These data suggest that in the initial period following loss of PRL-3, TNFα stimulates the TNF-R1 NF-ĸB program leading to senescence. After 24 h of sustained TNFα signaling, however, the TNF-R1 pathway shunts toward the initiation of cell-extrinsic apoptosis. This hypothesis would support our immunofluorescence observation of reduced p65 staining in PRL-3 knockdown cells treated with TNFα, suggesting downregulation of NF-ĸB to promote apoptosis.

### Blockade of the TNFα-associated TNF-R1 extrinsic death pathway confers resistance to cell death following PRL-3 knockdown

**Figure 5:**
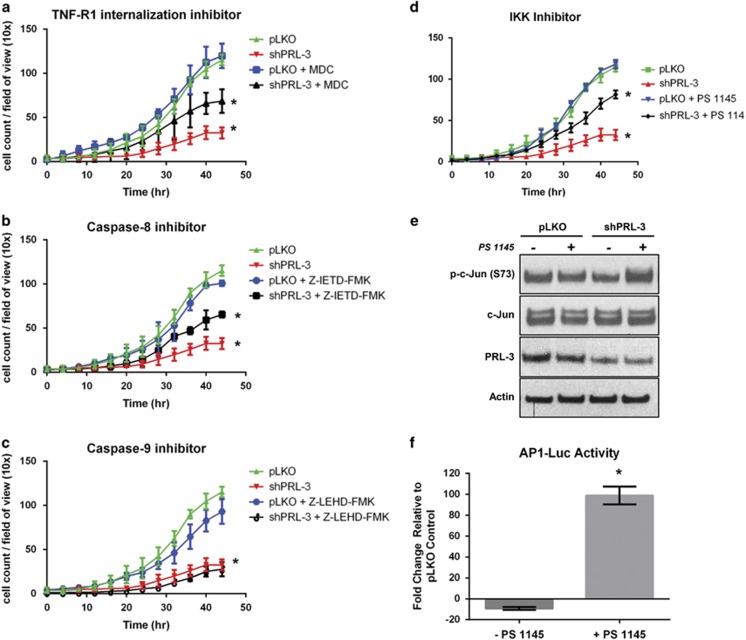
Blockade of the TNFα-mediated TNF-R1 extrinsic death pathway confers resistance to PRL-3 knockdown-mediated cell death, while inhibition of NF-ĸB following PRL-3 knockdown sustains TNBC cell survival through c-Jun/AP-1. Real-time kinetic monitoring of BT-20 cell proliferation as determined by the IncuCyte Zoom Live Cell Imaging System. Control cells (green) and PRL-3 knockdown cells (red) were treated with vehicle (blue) or the following inhibitors (black): (**a**) TNF-R1 internalization inhibitor (monodansylcadaverine (MDC), 100 μM), (**b**) Caspase-8 inhibitor (Z-IETD-FMK, 20 μM) or (**c**) Caspase-9 inhibitor (Z-LEHD-FMK, 20 μM). (**d**) BT-20 cell proliferation as assessed in (**a–c**) with IKK inhibitor (PS-1145, 10 μM). (**e**) Immunoblot analysis examining changes in activation of c-Jun assessed by phosphorylation of serine 73 in control (pLKO) and BT-20 cells with PRL-3 knockdown (shPRL-3) treated with PS-1145 (10 μM). (**f**) Fold change of AP-1 activity in PRL-3 knockdown BT-20 cells transfected with an AP-1-luciferase reporter plasmid and treated with PS-1145 (10 μM). Data represented are the mean±s.d. of three independent experiments. *P<0.05 as determined by Student *t*-test on final data point.

To investigate whether increased levels of TNFα following PRL-3 knockdown are responsible for promoting eventual cell death through TNF-R1, we blocked a critical step in the initiation of the extrinsic cell death pathway. TNF-R1 internalization is required for recruitment of TRADD, FADD and caspase-8 and activation of TNFα-induced apoptosis.^22^ The transglutaminase inhibitor, monodansylcadaverine, blocks TNF-R1 endocytosis and prevents TNF-induced apoptosis.^23^ We found that monodansylcadaverine treatment in PRL-3 knockdown cells conferred significant resistance to PRL-3 knockdown-mediated cell death (Figure 5a). Importantly, the effect of PRL-3 knockdown on TNF-R1-mediated cell death is not the result of reduced expression of either TNF-R1 or TNF-R2 as determined by qRT–PCR (Supplementary Figure 1). Moreover, inhibition of caspase-8, downstream of TNF-R1 endocytosis and responsible for activating extrinsic apoptosis, induced similar levels of resistance (Figure 5b). In contrast, blockade of the intrinsic cell death pathway using a caspase-9 inhibitor showed no resistance to PRL-3 knockdown-mediated cell death (Figure 5c), further validating that elevated TNFα levels following PRL-3 knockdown, eventually induces cell death through an extrinsic TNF-R1-guided pathway.

### Inhibiting NF-ĸB activation following PRL-3 knockdown sustains TNBC cell survival through c-Jun/AP-1

Our studies suggest that the NF-ĸB senescence program may be a pre-requisite step to TNFα-associated TNF-R1 cell death. Therefore, we hypothesized that blockade of the NF-ĸB signaling pathway following PRL-3 knockdown might result in accelerated cell death or sustained cell survival. We blocked NF-ĸB signaling using the IKK inhibitor, PS-1145, in BT-20 cells and observed sustained survival in cells exhibiting PRL-3 knockdown (Figure 5d).

To determine whether AP-1, a heterodimeric transcription factor composed of c-Fos and c-Jun and regulated by the TNF-R1 pathway,^24^ is responsible for the observed level of cell survival following PS-1145 treatment, we examined c-Jun activation in BT-20 cells. We observed substantial activation of c-Jun in cells with PRL-3 knockdown, and treated with the IKK inhibitor, as assessed by phosphorylation at serine 73 (Figure 5e). Moreover, to investigate the functional significance of c-Jun phosphorylation in the context of AP-1 transcriptional activity, we transiently transfected PRL-3 knockdown cells with an AP-1 luciferase construct and treated cells with the IKK inhibitor. We observed enhanced AP-1 transcriptional activity in BT-20 cells with combined PRL-3 knockdown and NF-ĸB inhibition (Figure 5f). These data suggest that bypass of the senescence program following PRL-3 knockdown does not accelerate cell death through TNF-R1, and thus, TNF-R1 is required for inducing apoptosis.

## Discussion

Cancer cell survival hinges on complex cell-intrinsic and cell-extrinsic stimuli that modulate cell cycle arrest, senescence, apoptosis and immune surveillance of cancer cells. Here, we demonstrate that expression of the metastasis-promoting phosphatase, PRL-3, is regulated by pro-inflammatory NF-ĸB signaling and that reduced PRL-3 expression results in a dynamic feedback loop modulating both the intrinsic cell cycle machinery and extrinsic TNF-R1 signaling pathway to control TNBC cell growth. The significance of these findings is underscored by a vital unmet need to understand molecular processes that promote the aggressive nature of TNBC, and the need to identify novel mechanisms for enhancing cancer cell death so that new therapeutic strategies may be explored.

We have discovered that knockdown of basal PRL-3 expression in TNBC cell lines results in activation of a cell cycle arrest and senescence program that cannot be rescued by mitogenic stimulation, thereby halting TNBC growth potential. In light of these findings, it is reasonable to propose that our observed function for PRL-3 is consistent with direct or indirect regulation of growth response factors in TNBC cells. This hypothesis is also consistent with the expected role for a member of the PRL family of phosphatases, initially discovered as immediate-early growth response genes following mitogenic stimulation in the regenerating liver.^25^

To explore the mechanism by which PRL-3 knockdown inhibits TNBC cell growth and promotes senescence, we examined the effects of PRL-3 expression on the cell cycle machinery. Interestingly, in normal, non-cancerous mouse embryonic fibroblasts, both overexpression and knockdown of PRL-3 have been shown to induce G1 cell cycle arrest.^16^ These data suggest that maintenance of basal PRL-3 levels is important for proper cell cycle progression in normal cells. Our data confirm that knockdown of PRL-3 expression in TNBC cells also promotes G1 cell cycle arrest through upregulation of either p16^INK4A^ or p27, depending on the mutational profile of the TNBC cell line. These data shed new light on how TNBC cells utilize alternative cyclin-dependent kinase inhibitors as a fail-safe mechanism to ensure G1 arrest following PRL-3 knockdown. Collectively, these data could be important in the context of a heterogeneous tumor with multiple aberrations impinging on the cell cycle checkpoint machinery and lend further rationale for targeting PRL-3.

Basak and colleagues^16^ observed PRL-3 overexpression in colon carcinoma or osteosarcoma cell lines failed to arrest cells in the cell cycle. Our data indicate that TNBC cells overexpressing PRL-3 have lost the ability to enforce cell cycle arrest or promote G1/S progression (data not shown). These data suggest that the phenotypic outcome of PRL-3 overexpression, above baseline levels in TNBC cells, does not promote growth but may execute other oncogenic functions such as driving migration, invasion and metastasis as we^6^ and others,^8^ have observed.

**Figure 6:**
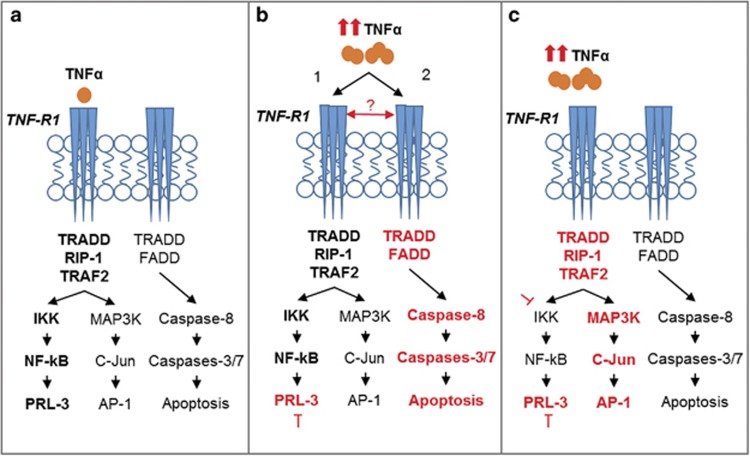
Working model of PRL-3 involvement in TNF-R1 pleiotropy. (**a**) PRL-3 is transcriptionally regulated by NF-ĸB in TNBC cells. (**b**) PRL-3 knockdown results in upregulation of TNFα RNA and protein leading to activation of the NF-ĸB pathway (pathway 1), coinciding with the period of cellular senescence. TNF-R1 is sensitized and activates an extrinsic apoptosis program (pathway 2) after prolonged TNFα upregulation following PRL-3 knockdown. (**c**) PRL-3 knockdown and blockade of the NF-ĸB pathway sustains cell survival through c-Jun/AP-1 activity.

We also sought to identify regulators of PRL-3 expression in TNBC cell lines to better understand the context in which cells utilize PRL-3. We identified the NF-ĸB pathway as a positive transcriptional regulator of PRL-3 expression (Figure 6a) in TNBC cells. This finding builds upon existing data indicating that constitutive activation of the NF-ĸB pathway promotes oncogenesis in ER-negative and hormone-independent tumors, such as TNBC.^26, 27^

To determine whether PRL-3 participates in a feedback loop with the NF-ĸB pathway, we knocked down PRL-3 and observed that senescence was accompanied by activation of NF-ĸB through TNFα upregulation (Figure 6b, pathway 1). Interestingly, accumulating evidence suggests a complex and context-dependent role for the NF-ĸB pathway in cancer, including the ability to maintain cellular senescence.^28, 29, 30^ PRL-3 was found to activate the NF-ĸB pathway in a TNFα-independent manner by interacting with RAP1,^31^ but how PRL-3 ablation leads to increased TNFα levels in TNBC cells remains poorly understood and warrants further investigation. Interestingly, Amar and colleagues^32^ also observed that administration of a PRL-3 peptide in mice can significantly attenuate adverse host responses to lipopolysaccharide stimulation, providing complete resistance to lethal doses of lipopolysaccharide owing to suppression of TNFα production. Although the exact mechanism promoting this finding was not determined, this study supports our observations of the inverse expression patterns of PRL-3 and TNFα.

An additional hypothesis is that NF-ĸB induces a senescence-associated secretory phenotype, which is characterized by a strong autocrine cytokine response that acts to achieve growth arrest.^28, 29, 30, 33, 34^ This idea is of particular interest because senescence-associated secretory phenotype has been shown to attract immune cells that eliminate senescent cancer cells. For example, we observed a modest increase in lymphotoxin-α in response to PRL-3 knockdown. Although generally thought to promote pro-survival signaling, a potential role of lymphotoxin-α in PRL-3 function is currently being investigated (Supplementary Figure 3). Examination of whether senescence-associated secretory phenotype ensues following PRL-3 knockdown and if this results in immune cell recruitment, activation and/or tumor cell clearance would provide valuable insight to the TNBC research community.

Because we observed a substantial increase in TNFα levels following PRL-3 knockdown, we also examined the longevity of the senescence program, reasoning that eventually, very high TNFα levels may be capable of activating cell death through an extrinsic, TNF-R1-mediated process (Figure 6b, pathway 2). This line of reasoning reveals several potentially interesting paradigms: (i) that the presence of TNFα, at low to moderate levels, confers resistance to apoptosis in TNBC cells and promotes PRL-3 expression and (ii) that a molecular sensor, such as the FADD-like apoptosis regulator, C-FLIP, may exist downstream of the NF-ĸB pathway to regulate signaling flux and control pleiotropism of the TNF-R1 pathway (Figure 6b, red arrow). Our findings demonstrate that PRL-3 knockdown results in TNBC cell sensitization to TNFα-induced apoptosis through TNF-R1. The crucial role of TNF-R1 in dictating the effect of PRL-3 knockdown on cell proliferation was determined by knocking down both PRL-3 and TNF-R1 together. We observed that loss of TNF-R1 expression reverses the anti-growth effects mediated by PRL-3 knockdown (Supplementary Figure 2). In addition, the TNFα-induced extrinsic cell death program cannot be accelerated by blocking the NF-ĸB senescence program that ensues immediately following PRL-3 knockdown (Figure 6c). This suggests that a sequential, temporally regulated process is required before TNFα can induce apoptosis in TNBC cells after impairing PRL-3 expression.

In summary, our data reveal a heretofore undescribed role for PRL-3 in TNBC biology. By engaging the adaptive signaling programs of the TNF-R1 pathway, loss of PRL-3 expression induces cellular senescence and upregulates TNFα to convert senescent TNBC tumor cells into apoptotic cells. This is a notably dynamic cellular response to reduced expression of an oncogenic phosphatase, particularly in the diverse background of genomic and proteomic alterations that are often associated with TNBC. Because high levels of PRL-3 expression are frequently observed in a variety of cancer types, our studies further demonstrate the potential utility of therapeutically targeting the PRL-3 signaling axis.

## Materials and methods

### Reagents

Recombinant human TNFα was purchased from PeproTech (Rocky Hill, NJ, USA; Cat. #300-01A), cycloheximide from Sigma-Aldrich (St Louis, MO, USA; Cat. #C-6255), monodansylcadaverine from Sigma-Alridch (Cat. #30432), caspase-8 and 9 inhibitors from BD Pharmingen (San Jose, CA, USA; Cat. #Z-IETD-FMK-550380 and Z-LEHD-FMK-550381), and the IKK inhibitor, PS-1145 (Cat. #4569) from Tocris Bioscience (Minneapolis, MN, USA).

### Plasmids, transfection and viral transduction

PRL-3 cDNA expression vector was purchased from Origene (Rockville, MD, USA; Cat. #SC308739). Transfections were carried out using Mirus TransIT LT1 reagent according to the manufacturer's instructions (Mirus Bio, Madison, WI, USA). Individual pLKO.1 lentiviral shRNA clones were obtained from the University of Colorado Cancer Center Functional Genomics Shared Resource. The RNAi Consortium identifiers are: TRCN0000010661 (shPRL-3 #1), TRCN0000355597 (shPRL-3 #2), TRCN0000014683 (shp65 #1), TRCN0000014684 (shp65 #2), TRCN0000359596 (shTNF-R1 #1) and TRCN0000359597 (shTNF-R1 #2). Transduced cells were selected in medium containing 2.5 ug/ml puromycin. PRL-3 shRNAs were previously validated.^6^

### Luciferase assays

The luciferase reporter used to measure NF-ĸB activity contained four copies of the NF-ĸB consensus sequence fused to a TATA-like promoter (P_TAL_) region from the Herpes simplex virus thymidine kinase promoter (pNF-ĸB-Luc, Clontech, Mountain View, CA, USA; Cat. #6053-1). The reporter used to measure AP-1 activity (pAP1(PMA)-TA-Luc) was purchased from Clontech (Cat. #6056-1). Reporter plasmids were transfected at 1 μg/2 × 10^5^ cells. Twenty-four hours after NF-ĸB-Luc transfection, cells were washed with PBS and lysed in luciferase harvest buffer. Twenty-four hours after AP-1-Luc transfection, cells were treated with 10 μM PS-1145 overnight then lysed in luciferase harvest buffer the next day. Luciferase assays were carried out as previously described.^35^ For TNFα neutralization studies, anti-TNFα antibody (Cell Signaling, Danvers, MA, USA; Cat #7321) was added at a concentration of 10 ng/ml.

### Quantitative RT–PCR

Total RNA isolation, cDNA synthesis and qRT–PCR were performed as previously described^35^ with the following oligonucleotides: PRL-3 5′-AGTTGCCCGCTTTACTTTGGTTGG-3′ and 5′-AGGAAGCTGCCCACTGTTTGGATA-3′, p65 5′-TATCAGTCAGCGCATCCAGACCAA-3′ and 5′-AGAGTTTCGGTTCACTCGGCAGAT-3′, TNFα 5′-AATCGGCCCGACTATCTCGACTTT-3′ and 5′-TTTGAGCCAGAAGAGGTTGAGGGT-3′, Cyclin E1 5′-GTACTGAGCTGGGCAAATAGAG-3′ and 5′-GAAGAGGGTGTTGCTCAAGAA-3′, Cyclin D1 5′-CCACTCCTACGATACGCTACTA-3′ and 5′-CCAGCATCTCATAAACAGGTCA-3′, E2F1 5′-GCTGGACCACCTGATGAATATC-3′ and 5′-GTCTGCAATGCTACGAAGGT-3′, p53 5′-AGGGATGTTTGGGAGATGTAAG-3′ and 5′-CCTGGTTAGTACGGTGAAGTG-3′, p27 5′-CTAACTCTGAGGACACGCATTT-3′ and 5′-TGCAGGTCGCTTCCTTATTC-3′, p16 5′-CGCTAAGTGCTCGGAGTTAATA-3′ and 5′-CGACCCTGTCCCTCAAATC-3′.

### Cell culture, senescence and immunoblot analysis

Cell lines were obtained from the University of Colorado Cancer Center Tissue Culture Shared Resource. All cells were authenticated by short tandem repeat DNA profiling performed by the UCCC DNA Sequencing and Analysis Core and determined to be free of mycoplasma contamination using the MycoAlert Mycoplasma Detection kit from Lonza (Walkersville, MD, USA; Cat. #LT07–218). β-Galactosidase staining was performed according to the manufacturer's protocol (Cat. #9860S, Cell Signaling). Immunoblots were performed according to our previous protocol.^36^ PRL-3 (Cat. #ab82568, Abcam; Cambridge, MA, USA), p65 (Cat. #8242, Cell Signaling), TNFα (Cat. #8301, Santa Cruz Biotechnology, Dallas, TX, USA), Phospho-c-Jun S73 (Cat. #3270, Cell Signaling), c-Jun (Cat. #9165, Cell Signaling), LTa (Cat. #MAB1370, R&D Systems, Minneapolis, MN, USA), β-actin (Cat. #A5441, Sigma-Aldrich). Immunofluorescence staining was performed as described^37^ using p65 antibody (Cat. #06–418, EMD Millipore, Billerica, MA, USA).

### Cellular size, count and proliferation assays

Cellular proliferation was assessed using the CellTiter 96 MTS cell Proliferation assay (Cat. #G109A, Promega, Madison, WI, USA). Samples were read at 490 nm in a Synergy 2 microplate reader (BioTek, Winooski, VT, USA). Cellular size, count and proliferation rates were also determined using the IncuCyte Zoom Kinetic Live Cell Imaging (Essen BioScience, Ann Arbor, MI, USA). IncuCyte cell recognition software calculated values based on cellular size over time, cellular count per field of view or percentage of cell confluence over time.

### Chromatin immunoprecipitation assay

p65 binding to the PRL-3 promoter was analyzed following 30 min of TNFα treatment in BT-20 and MDA-MB-468 cells. The chromatin immunoprecipitation assay was performed as previously described.^38^ The purified DNA was then subjected to quantitative real-time PCR using oligonucleotides specific for each of 5 putative p65-binding elements in the human P3 promoter (−7 kb, −6 kb, −4 kb, −1.3 kb, +0.4 kb). Oligonucleotides specific to the NF-ĸB target gene promoter of IL-6 were used as a positive control. Oligonucleotides: Site 3 5′-CTTAGCTTGAGCCCTCCCAC-3′ and 5′-CCCCTCTCCTGAGCTCCCAG-3′, Site 4 5′-CTTAGCCAGGCCCAGTGGGC-3′ and 5′-GCTGACTCCAGGGCAGGAAC-3′, Site 5 5′-CCGCTCGCTCCCGCTGTTAC-3′ and 5′-GAAGGATCCCGGAACGCTCC-3′, Site 6 5′-GAGAGAACCCAGTTAACTGG-3′ and 5′-CAAGCCCTGCAGAACCCTTC-3′, Site 7 5′-GTGACCCCTGTGGAGTGGAT-3′.

### Cell cycle and apoptosis analysis

Cells were incubated in Krishan stain overnight and analyzed using a Beckman Coulter (Brea, CA, USA) FC500 flow cytometer. Doublets are excluded from the analysis using the peak vs integral gating method. ModFit LT software (Verity Software House, Topsham, ME, USA) was used for cell cycle analysis. CellPlayer 96-well Kinetic Caspase-3/7 reagent was used at 5 μM according to the manufacturer's instructions (Essen Bioscience).

### Statistical analysis

Data are expressed as mean±s.e.m. Significance was calculated using the Student *t*-test in GraphPad Prism version 6.04 software. Tests were two-sided with significance set at *P* <0.05.
