# Supplementary material for: Loss of the oncogenic phosphatase PRL-3 promotes a TNF-R1 feedback loop that mediates triple-negative breast cancer growth
Source: Oncogenesis. 2016 Aug 15;5(8):e255–. doi: 10.1038/oncsis.2016.50 (PMC5007826; doi:10.1038/oncsis.2016.50)
Supplement: Supplementary Figures Legends [file oncsis201650x1.docx]

**SUPPLEMENTARY FIGURE LEGENDS**

**Supplementary Figure 1.** Validation of PRL-3 and TNF-R1 knock down by qRT-PCR. Bar graph depicts changes in mRNA expression for PRL-3, TNF-R1, TNF-R2, TNFa and LTalpha (LTa) in MDA-MB-468 cells following stable viral transduction with non-silencing control shRNA (pLKO; blue), one shRNA to PRL-3 (shPRL-3; green), two shRNA clones to TNF-R1 (shTNF-R1-1; orange and shTNF-R1-2; brown). Also shown are the changes in mRNA expression for the same genes following co-transduction of MDA-MB-468 cells with an shRNA to PRL-3 and both shRNA clones to TNF-R1 (shPRL-3 + shTNF-R1-1; purple, shPRL-3 + shTNF-R1-2; dark green). Total RNA isolation, cDNA synthesis and qRT-PCR were performed as previously described^35^ with the following oligonucleotides: PRL-3 5’-AGTTGCCCGCTTTACTTTGGTTGG-3’ and 5’-AGGAAGCTGCCCACTGTTTGGATA-3’, TNF-R1 5’-CTCCAAATGCCGAAAGGAAATG-3’ and 5’-ATAATGCCGGTACTGGTTCTTC-3’, TNF-R2 5’-TGCATCGTGAACGTCTGTAG-3’ and 5’-GGAATCTGTGTCTCCCATTGT-3’, TNFα 5’-AATCGGCCCGACTATCTCGACTTT-3’ and 5’-TTTGAGCCAGAAGAGGTTGAGGGT-3’, LTα 5’-AGATCCACACACAGAGGAAGA-3’ and 5’-CCCTCTCTCCATCCTCCATAAA-3’. Data represented is the mean ± SD of three independent experiments. * = p-value <0.05 as determined by Student *t*-test.

**Supplementary Figure 2.** Blockade of the TNF-R1 extrinsic death pathway by knock down of TNF-R1 confers resistance to PRL-3 knock down-mediated cell death. Real-time kinetic monitoring of MDA-MB-468 cell proliferation as determined by the IncuCyte Zoom Live Cell Imaging System. MDA-MB-468 cells were transduced with non-silencing control shRNA (pLKO; blue), shRNA to PRL-3 (shPRL-3; green), or two different shRNA clones to TNF-R1 (shTNF-R1-1; orange and shTNF-R1-2; brown). Cells were also co-transduced with shRNA to PRL-3 and either clone of TNF-R1 shRNA (shPRL-3 + shTNF-R1-1; purple and shPRL-3 + shTNF-R1-2; dark green). Data represented is the mean ± SD of three independent experiments. * = p-value <0.05 as determined by Student *t*-test on final data point.

**Supplementary Figure 3.** Administration of an anti-LTα antibody confers partial resistance to PRL-3 knock down-mediated cell death. Real-time kinetic monitoring of MDA-MB-468 cell proliferation as determined by the IncuCyte Zoom Live Cell Imaging System. MDA-MB-468 cells were transduced with non-silencing control shRNA (pLKO Ctl; blue) or shRNA to PRL-3 (shPRL-3 Ctl; orange). The same cells were then treated with a neutralizing antibody to LTα to examine the effect of blocking the activity of LTα (pLKO + aLTa; green and shPRL-3 + aLTa; brown). Data represented is the mean ± SD of three independent experiments. * = p-value <0.05 as determined by Student *t*-test on final data point.
