# Supplementary figures and images for: Loss of the oncogenic phosphatase PRL-3 promotes a TNF-R1 feedback loop that mediates triple-negative breast cancer growth
Source: Oncogenesis. 2016 Aug 15;5(8):e255–. doi: 10.1038/oncsis.2016.50 (PMC5007826; doi:10.1038/oncsis.2016.50)

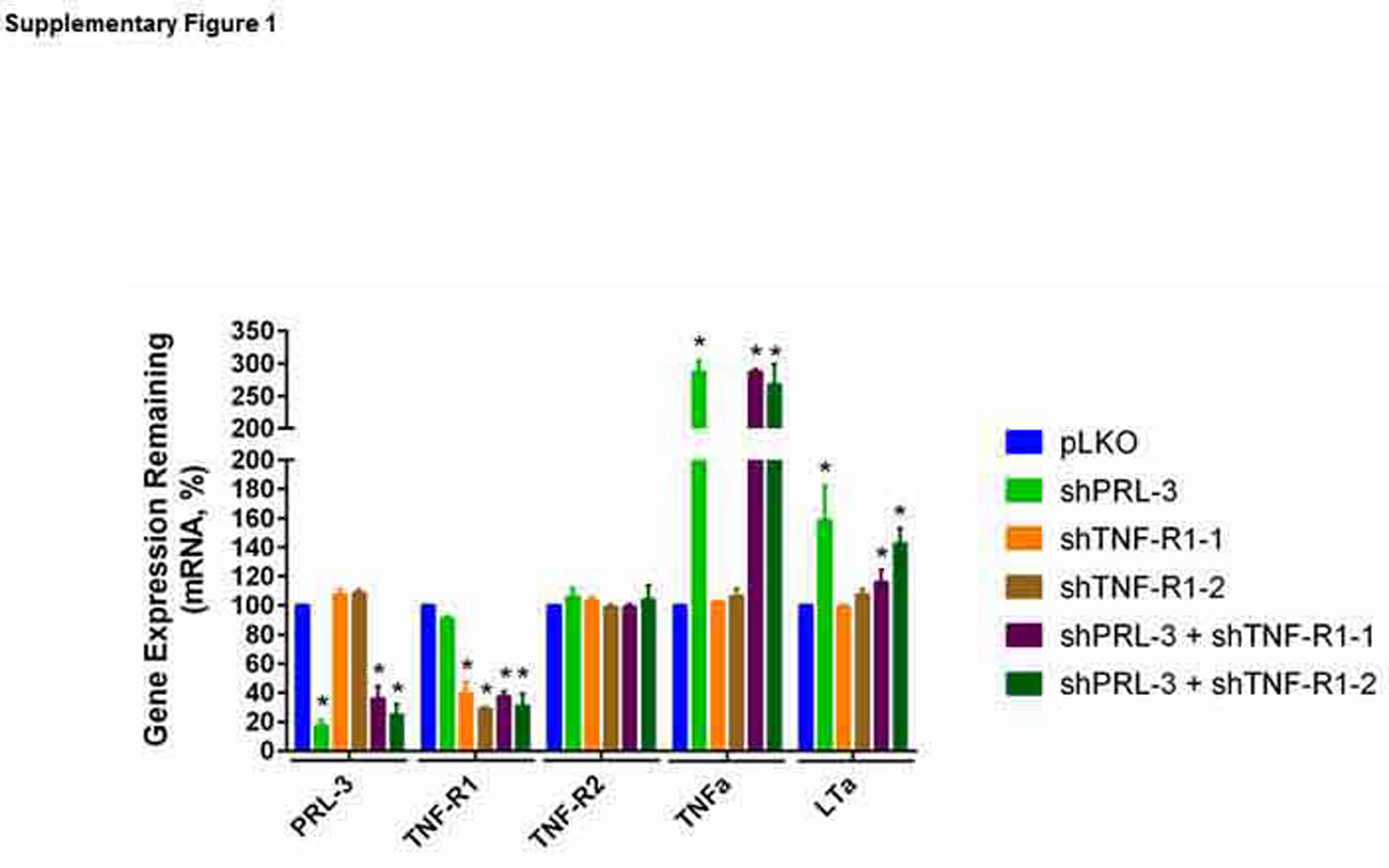

Supplement: Supplementary Figure 1 [file oncsis201650x2.tif]

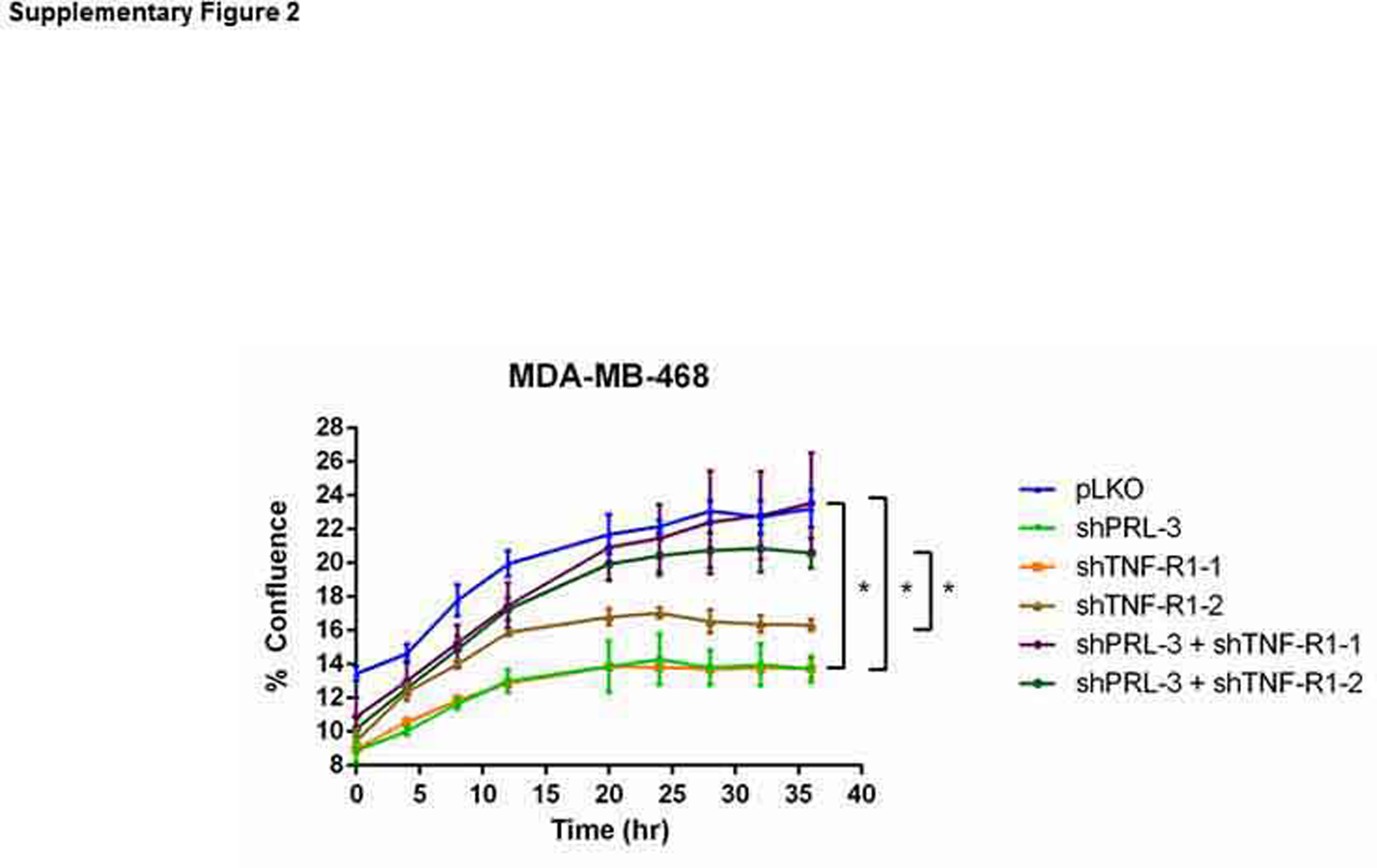

Supplement: Supplementary Figure 2 [file oncsis201650x3.tif]

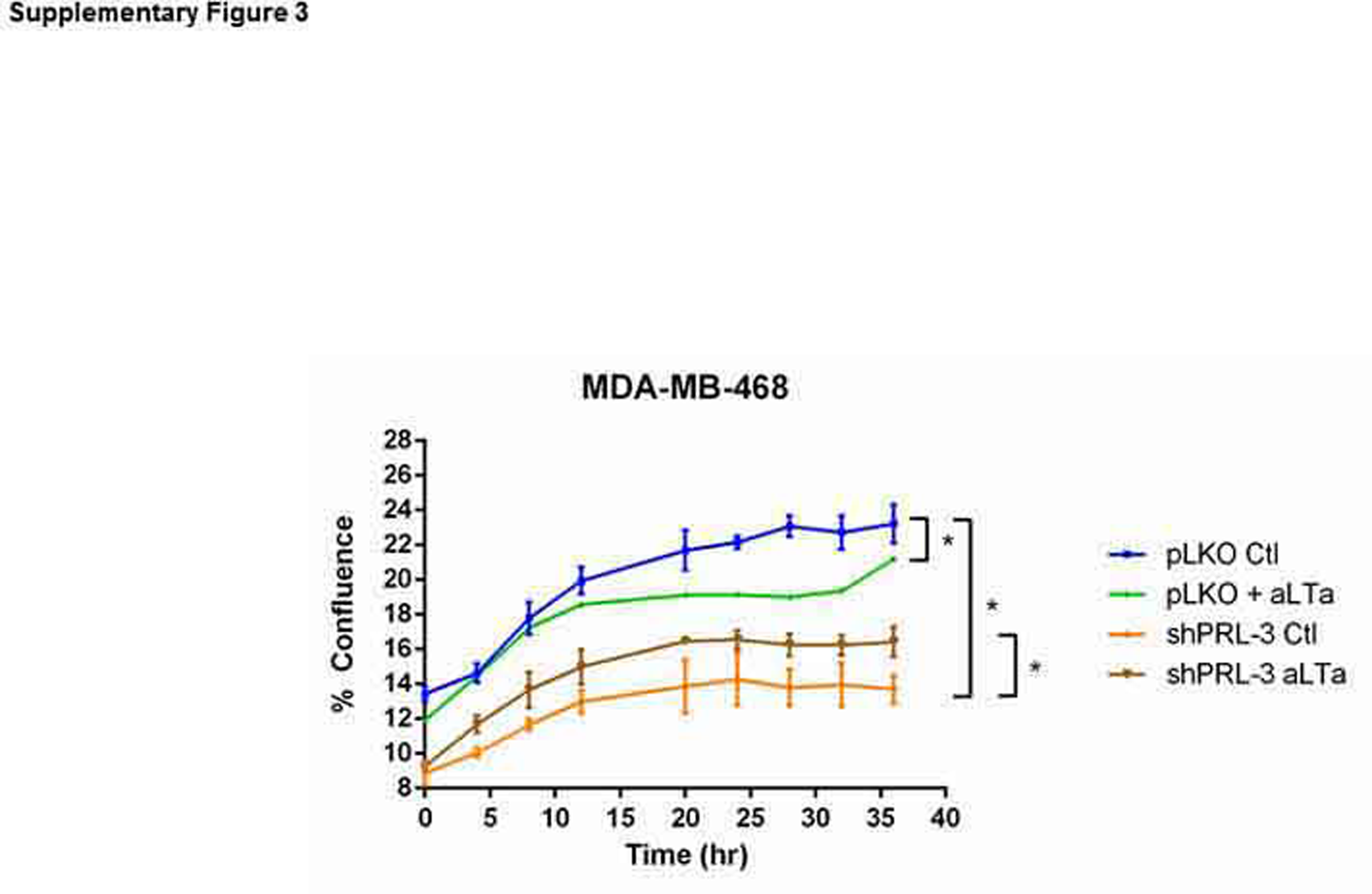

Supplement: Supplementary Figure 3 [file oncsis201650x4.tif]
